# Plastic Surgery, Critically Examined as to What It Has Hitherto Accomplished; a Prize Essay of the Medical Society of Gent

**Published:** 1845-04

**Authors:** 


### Art. VI.

1. *Die Plastische Chirurgie nach ihren bisherigen Leistungen kritisch dargestellt. Eine von der Medicinischen Gesellschaft zu Gent gekrönte Preisschrift.* Von Dr. Friedrich August v. Ammon, Hofrath, Leibarzt s. m. des Königs von Sachsen, &c. &c.; und Dr. Moritz Baumgarten, Practishem Arzte zu Dresden, &c.—*Berlin*, 1842.

*Plastic Surgery, critically examined as to what it has hitherto accomplished; a Prize Essay of the Medical Society of Gent.* By Dr. F. A. von Ammon and Dr. Moritz Baumgarten.—*Berlin*, 1842. 8vo. pp. 310.

2. *Traité sur l'Art de restaurer les Difformités de la Face, selon la Méthode par Déplacement, ou Méthode Française.* Par M. Serre, Professeur de Clinique chirurgicale à la Faculté de Médecine de Montpellier, &c. &c.—*Montpellier et Paris*, 1842.

*Treatise on the Art of Restoring Deformities of the Face, by displacement (sliding of the flap), or the French method.* By M. Serre, Clinical Professor at Montpellier, and Surgeon to the Hospital St. Eloi.—*Montpellier and Paris*, 1842. 8vo. pp. 468.

3. *Cases of Deformities of various kinds, successfully treated by Plastic Operations.* By Thomas D. Mütter, m.d. Professor of Surgery in Jefferson Medical College, &c.—*Philadelphia*, 1844. 8vo. pp. 38.

4. *Cases of Deformity from Burns, successfully treated by Plastic Operations.* By T. D. Mütter, m.d. &c.—*Philadelphia*, 1843. pp. 24.

5. *A Report on the Operations for Fissures of the Palatine Vault.* By T. D. Mütter, m.d. &c.—*Philadelphia*, 1843. pp. 28.

As six years have now elapsed since we introduced the subject of Plastic Surgery to our readers, during which interval there have been many active labourers in this division of the field of medicine, we have thought that the time has arrived for us to go forth and see what of novelty and interest we may glean for the profit and entertainment of those who honour us by their confidence. We are further urged to this task by a promise which we recently made of renewing our acquaintance with Dr. Mütter's pamphlets at an early period, as well as by a consciousness of the peculiar bareness of our own literature in this useful department of surgery.

The reason of this bareness we are at a loss to comprehend, as indeed are likewise our continental brethren; for we find Von Ammon speculating why a people of so practical a turn as the English should not have given some more substantial evidence of their cultivation of plastic surgery, than such as is to be found scattered here and there in our journals, or forming a scanty chapter in our systematic works of surgery. The love of novelty, however, is probably not so great with the English as with either the Germans or French, nor are we so ready to give an undue importance and weight to that which is new, until it has received the sanction of time and experience. Be this as it may, it certainly does excite our surprise that the last six years have not elicited any work embodying the experience of some one of our countrymen possessing the requisite opportunities and taste for the art; and we therefore the more willingly apply ourselves to the task of supplying, in some sort and measure, the deficiency.

Whilst we must admit that the art of which we now treat is one of a purely mechanical nature, which indeed is implied in its name, we feel ourselves justified in claiming for it, on the score of its usefulness and the relief it affords to human suffering, a more elevated rank than it would at first seem to merit. "La Chirurgie plastique peut devenir la fleur de toute la médecine opèratoire," was the motto selected by Von Ammon for his treatise; and this prettily as well as justly embodies the sentiment with which he, in common with other writers on the subject, regards this favorite branch of the healing art; healing in its most extended sense, as well to the wounded vanity of the deformed outcast from society, as to the physical suffering of the blear-eyed and lipless and mouthless wretches, whom it restores to those blessings, which ruthless disease, or, perchance, the self-assassin's hand has deprived them of, marring at once both the usefulness and beauty of that fairest of created things, the human face. Further, we would claim consideration for plastic surgery, on the ground of its being a division of our art requiring considerable natural ingenuity, as well as acquired skill; and we venture to assert that there are comparatively few who possess the requisite qualifications to become expert plastic operators, that is, in the more complex operations embracing (if we may credit the authors) apparent impossibilities. Such plastic operations as may be reduced to rules, the Rhinoplastic for example, require little more than a strict adherence to these rules for their proper performance; but in a great many the operator has to form as well as to carry out his own design, by which his ingenuity as well as skill is tested: and he has also to calculate how far he may trust to the cooperation of Nature, or where she is likely to take offence and forsake him; leaving, in such case, his patient in a worse condition than before he was interfered with. Therefore, though the part the surgeon takes is purely mechanical, it is requisite he should bring with him all the knowledge which can bear upon the probability of retained vitality and adhesion of parts, in the new relation which they are forced to bear to each other.

In our last article* we gave our readers a succinct history of the origin and progress of plastic surgery from the earliest period of its existence until the present day, and also entered somewhat at large upon the various divisions of the art. We shall, of course, avoid retracing our steps, and therefore request our readers to regard the present article as supplying the deficiencies which the former presented, together with such new matter as we may find worthy of transcription from the works which head this paper.

The authors whom we have selected as representatives of their several countries have for some time been favorably known, as surgeons, to the profession, and we therefore have the more confidence in the results which their labours in this peculiar department present us with. Von Ammon and Mütter had already earned a reputation as plastic surgeons when we first took up the subject in our Journal, as a reference to the article in question will show: and M. Serre's 'Traité de la Réunion immédiate' constituted, as he informs us in his preface it did, a fitting introduction

* No. for April, 1839.

to the study of the restoration of lost parts; and he accordingly devoted especial attention to this subject. The conjoint treatise of Von Ammon and Baumgarten gained for them the prize offered by the Medical Society of Ghent, and affords a good view of the state of plastic surgery in all its departments; though, as might be anticipated, the peculiar views of the authors and their own operations hold a prominent position ; a remark which is equally applicable to their French competitor. We had occasion in our former review to complain of the rather uncourteous handling which his Gallic contemporaries received at the hands of Zeis; this is now paid back with interest by M. Serre, who acts the part of champion to his nation (not forgetting his own peculiar self,) and employs invective as well as reclamation in a way which, to say the least, is undignified, and savours much both of bad taste and want of temper, alike subjecting its author to the charge of jealousy, and interfering with the usefulness of his work. The simple details contained in the unobtrusive pamphlets of Dr. Mütter,* at once stamp him as an enterprising and clever surgeon, who is conscious of possessing merit and originality enough of his own, to spare their due meed to others who have laboured in the same field with himself.

A prominent and laudable feature in M. Serre's volume is the effort he makes to reduce, as much as possible, the art of remedying defects by plastic operations to fixed principles and rule; and he especially combats the opinion of Velpeau that cheiloplasty is unsusceptible of detailed directions, and that the operator must modify his plan of operating almost as often as he practises it: how far he succeeds in making good his position we shall give our readers some opportunity of judging. Another and even more prominent object of our French author is to claim for his country a certain mode of operating which he distinguishes as "la méthode par déplacement, *ou* la méthode Française." He accordingly states in his preface that it is his intention to treat neither of the Indian nor Italian methods of operating, but that the theme of his discourse and praise will be the French mode. Now we cannot but protest in the outset against this assumption on the part of M. Serre ; it may be flattering to his national vanity thus to appropriate for his own country that which is certainly now admitted to be the most useful and generally applicable form of operation ; but, if we interpret the word aright, (and that we have ample means of doing by the subsequent descriptions,) it is one which has been extensively employed heretofore in Germany as well as France. That M. Serre has successfully answered the objection that plastic operations are attended with considerable hazard to the patient, is well attested by his statement, that of one hundred and fifty cases which have fallen under his own immediate care, only one was lost, and that from a casualty not necessarily associated with the operation. This large share of practice will dispose us to pay that respect which is justly due to the opinions of the operator ; whilst we cannot but smile at the closing paragraph of his preface, that "he has it at heart to prove that, contrary to the assertions of MM. Zeis and Phillips, as regards plastic surgery of the face, France need not be jealous even of Germany ;" which national "amour propre" the author

* First published in the American Journal of Medical Sciences.

freely confesses would alone have prompted him to publish the present work.

Having extended the foregoing remarks beyond what was our original intention, though we think not beyond what is necessary as an introduction to our authors, and our duty as reviewers, we are now prepared to commence the analytical division of our labours, which we shall do by passing at once to the details of the various divisions of plastic surgery, collating as we proceed. Our table of contents will therefore include the following heads: Operations connected with the Nose, Mouth, Cheeks, Lips, and Eyelid; and we shall subsequently devote a few remarks to the other pamphlets of Dr. Mütter, named at the head of this article.

I. The nose. In the *rhinoplastic* operation we have not a great deal of novelty to record. Von Ammon describes the various operations which have been performed for the removal of the whole or part of the nose, commenting on their relative advantages. The principal points of importance which he insists on have been pointed out in our previous article, and we therefore do not consider it necessary here to repeat them. He notices Labat's operation for the formation of a new septum from the palm of the hand, but objects to it as calculated to maim the thumb and produce, after all, but a poor substitute for the original structure: when practicable he prefers taking the necessary material from the upper lip,—a preference in which we entirely concur. In his chapter on the same subject M. Serre narrates a case of Professor Dieffenbach, which escaped our notice in our previous review of his work, and which we are tempted to notice from the boldness and ingenuity with which it was conceived, and the success which attended it. The subject of the operation was a young girl of twelve years of age, whose face was sadly disfigured by scrofula, the centre presenting in place of a prominent nasal organ, " a tortuous irregular furrow which gave the face the appearance of a death's head:" a portion of the nasal tegument covered in the empty space left by the loss of bone; but this was folded inwards. The following is the account of the operation:

" Two incisions were made on the sides of the depressed nose, throughout its whole length, and extending from below upwards; by this an isolated band of skin was left, three times as broad at its base as at its upper part, and united to the upper lip by a fillet of skin both thin and short: above, this band adhered to the integument by a narrow bridge. The soft parts were then separated at the two extremities of the nose down to the bone. The next step was, by means of two semilunar incisions, continuations of the lateral incisions, to separate the alæ nasi from their external adhesions; and the operator was thus enabled to draw out, from the depth at which they had been so long hidden, the bands of skin, contracted above but broader below, which resulted from the incisions above enumerated. M. Dieffenbach lastly excised the internal margins of the back of the nose (bounding the furrow), and the external edges of the lateral borders and alæ, the firmness and thickness of which encouraged his hope of success. After a short interval the opposed borders, which were destined to form the back of the nose, were first connected by six points of suture; and then in like manner the alæ and lateral margins were attached to the skin of the cheeks and upper lip by eight sutures, the cheeks having been previously prepared by separation from the bone to the extent of some lines. The band which formed the septum being found too short, was elongated by two small lateral incisions in the upper lip; and a small quill enveloped in oiled charpie was introduced into either nostril. Further, with a view to preventing the approximation of the cheek and corresponding border of the nose, a long and narrow needle, furnished with a piece of rounded leather at its extremities, was passed from the integuments of the cheek to the middle of the nose, and its point rolled round with pincers in a spiral form." (Serre, p. 236 et seq.)

It is unnecessary for us to follow out the history of this case further in detail: suffice it to say that this resuscitated organ gradually assumed more and more consistence, during which it was supported, for the first ten days as we have described, and afterwards for an equal time by a needle on either side; that cauterization of the deep surface of the nose, and frequent injections were employed; and lastly that the upper lip was made to aid in completing the septum. The result, as we have already remarked, was entirely satisfactory.* M. Serre closes his comments on this case by claiming for M. Larrey the merit of a similar operation ten years previously; in proof of which a case is appended of a somewhat similar process for remedying a like deformity; but as we are by no means disposed to act the part of umpire in this instance, we leave our readers who may be interested in the subject, controversially considered, to consult for themselves the original. The concluding flourish is all we can find room for: "Let M. Zeis then cease to set himself up against M. Larrey, by saying that he has only united some wounds of the nose by retentive bandages."

In our former article we spoke of the various expedients resorted to for the formation of a new fleshy septum to the nose, and especially approved of that operation which, by removing the superabundant texture from the upper lip, at once improved its appearance and supplied the requisite material for the neighbouring organ. We see that M. Serre proposes what he considers an improvement on this operation, which consists in "detaching the flap by its superior part, and subsequently separating it after it has contracted the requisite adhesion to the tip of the nose." We are disposed to think that the closure of the hiatus in the lip would be found much more difficult in this mode of operating; and the ready conversion of mucous membrane into epidermis renders it superfluous. We perceive, by the way, that, in connexion with this division of the subject, M. Serre attributes considerable merit to M. Blandin for the first application of the principle which involves the convertibility of the mucous membrane above noticed; the memoir alluded to bears the date 1839 to 1840,† that the fact and its application were familiar to us in the early part of the former year our readers may satisfy themselves by reference to p. 403 of our Seventh Vol.

M. Serre's twenty-first case presents us with a good specimen of the satisfactory results obtained by successive operations,—in this instance seven in number. In the individual in question (a young woman) "the greater part of the left cheek was destroyed; a considerable portion of the superior maxillary bone, and the corresponding soft parts to a still larger extent were involved in this solution of continuity, which established a permanent communication with the mouth, of which the boundaries were, internally the median line of the face, externally an imaginary line extending vertically from the angle of the eye to the lower jaw, in-

* The case was first communicated to the French institute in 1830.
† Serre, Note to p. 258.

feriorly the lower lip, and superiorly a horizontal line five or six lines removed from the lower border of the orbit." Here was indeed a test for plastic surgery: the operations extended over a twelvemonth, and were ultimately so entirely successful as to call forth the eulogy of the operator (M. Roux,) in the following words: "After such a triumph obtained by the plastic art, I should be tempted to maintain that there is nothing beyond the range of possibility referring to restoration of the face." The date of this operation (for the details of which we have not room,) was as early as 1826. We must find space, however, for a case of M. Lisfranc's, in which the rhinoplastic operation was performed by the French method; it is as follows:

"In the month of June 1830, a woman, aged 30 years, entered the Hospital of la Pitié, with an almost total loss of the nose, the position of which was occupied by a hideous opening, which left uncovered all the anterior parts of the nasal fossæ. The neighbouring callous and adherent skin was folded inwards and continuous with the mucous membrane; whilst the septum, in part destroyed, fell towards the right side, and totally obstructed the corresponding nostril. To remedy this defect, M. Lisfranc conceived the idea of detaching from each side of the nose a triangular flap of skin, the base of which was directed towards the mouth, whilst the summit remained attached, and being destined to serve as a pedicle, would correspond to the inner angle of the eye. These two flaps were to be dissected up, and being approximated, were then to be united in the median line of the nose (that was to be). The first step of the operation was to reestablish the anterior opening of the right nostril, which was effected by dividing with a bistoury the adhesions which produced the obliquity of the septum. The requisite incisions were then carried right and left on the cheeks: the first extended from three lines beneath and internal to the inner angle of the eye downwards and outwards, to the level of the inferior border of the nasal aperture. The remains of the folded and adherent skin were then dissected up from around this cavity, and were made even and pared in preparation for their union in the median line. A second incision then passed transversely from the inferior extremity of the former to the pared edges of the cicatrized margin, and separated the base of the flap from the upper part of the lip, and facilitated the dissection of this triangular flap of skin The same manipulation was repeated on the opposite side, and after tying several small arteries, the two resulting flaps were united to each other by three points of suture, and served to form the end of the nose. The next step was to reunite the cheeks at the external border of each flap, in doing which it was found necessary to curtail the breadth of the lip by the excision of a triangular portion right and left. This part of the operation was effected by two incisions, of which one extended from the point of termination of the lateral incision obliquely downwards and outwards, and the other from some lines internal to the bleeding margin of the upper lip, and joined the termination of the first. Lastly, the cheeks were dissected up to a sufficient extent to favour their approximation to the external borders of the flaps, to which they were attached by three points of suture: two other points fixed the edge of the upper lip to the corresponding part of the cheek, and the operation was completed, with the exception of the formation of the septum, which was deferred till a later period." (Serre, p. 284 et seq.)

We have thought it right to give the details of the above operation, as illustrative of the 'méthode par déplacement' so much eulogized by M. Serre, not because we think it an improvement on the Indian operation, but to give our readers an opportunity of judging for themselves. We must acknowledge we should have anticipated, before looking to the end of the case, that the great drawback to which this operation would be obnoxious must be the necessary flattening, from the want of support, which is in a measure given by the twisted pedicle of the flap derived from the forehead, but more especially from the necessary after-contraction resulting from the loss of soft parts in the cheek. Let us turn then to M. Serre's comment on the case:

"I am not," he says, "disposed to speak of M. Lisfranc's operation as exempt from all difficulty: I even feel it a duty to declare that the operation was only attended with partial success, and that the new nose has gradually become flattened."

He further suggests that "secondary corrections" might in such cases be advantageously had recourse to. And then, on the other hand, our attention is called to the opinion of M. Velpeau, that the Indian method is a dangerous operation, not unfrequently terminating fatally, as proved in the practice of Lisfranc and Dieffenbach, the latter of whom lost two out of six patients during his stay at Paris. We are not aware of such disastrous consequences having occurred to any of our countrymen; at least, all the cases which have come under our cognizance have been attended by partial or complete success; or at worst, by sloughing of the new-made organ. Before leaving this part of our subject, we cannot refrain from noticing a candid remark of M. Serre's, in which most impartial judges must concur:

"I am acquainted with the successive improvements which Delpech, Dieffenbach, Lisfranc, Blandin, Labat, and many others have introduced in the mode of performing the rhinoplastic operation; but I cannot the less persist in saying that it appears to me there is exaggeration in all that has been written relative to the regularity of new noses, whatever may be the method adopted for making them. For my part, I have made my share, and I am not afraid to publish that I have never obtained, in this respect, such satisfactory results as I could wish." (p. 271-2.)

II. The mouth. The mouth-making department of our subject receives but a brief notice at the hands of our German authors, who appear entirely satisfied with the clever operation of Professor Dieffenbach, already detailed by us. The particulars of Dr. Mütter's case, in which he followed the Berlin Professor's directions are narrated in his pamphlet; and the satisfactory conclusion of the case was considerably enhanced by the entire failure, of the previous attempts, to remedy the defect by simple lateral incisions, although "tents were introduced to prevent the lips of the wounds from uniting. This appeared at first to be productive of some good, but in a short time they cicatrized and contracted, and the patient remained in as uncomfortable a condition as before." M. Serre likewise has a panegyric on this operation, which he acknowledges himself "forced to admit as the type of all that plastic surgery can offer of the ingenious and delicate;" yet he considers it as not without its drawbacks and inconveniences, which preamble of course prepares us (as usual, we are compelled to say,) for some criticism on and variation of his German contemporary's mode of proceeding. In the first place the idea of the operation is attributed to Werneck, who, it appears from Von Ammon's account, first proposed and adopted the plan of uniting mucous membrane and skin in extension of the mouth by lateral incisions, as early as 1817: in fact his operation was the same as that more recently practised by Diffenbach. But was M. Serre ignorant that the case in question was not published until 1830, and therefore cannot detract from the merit of originality due to the Berlin professor, whose operations were performed in the interim? If aware of this it is scarcely ingenuous or liberal to have concealed it. But now for the variation in the operation, which, if as successful, we are willing to accept as an improvement on account of its greater simplicity. It consists in a simple lateral incision, on either side of the mouth by which the aperture is extended ; and the reunion without loss of structure, of the opposed borders of skin and mucous membrane : where the thickness and induration of the intervening tissues require it, a slice (tranche) of the soft parts may be removed to facilitate the completion of the operation by the application of the necessary sutures. This operation has likewise succeeded in the hands of M. Velpeau ; the only advantage which the German has over this modification is, the rounded coral margin which the greater abundance of mucous membrane must give more perfectly.

III. The cheek. The *meloplastic* operation, by which the partial loss of the cheek is renewed, constitutes a division of plastic surgery in which the so-named French method is peculiarly applicable. Von Ammon describes the operations of Graefe, Roux, and Dieffenbach, with criticisms upon each; and M. Serre deprecates the employment of measures which require any twisting of the pedicle of the flap ; but as all these operations where loss of structure is involved, must be necessarily subject to modifications varying with the deficiency to be replaced, we shall pass by the comments and details of our European continental authorities, and confine ourselves to the illustration of what plastic surgery can do in this department by citing one of Dr. Mütter's cases.*

"In the month of March, 1842, A. T——, aged 30, applied to me for the relief of a distressing deformity, occasioned by the abuse of mercury. About six years before I saw her she had been most severely salivated for a bilious fever, and in consequence of ulceration attacking the right cheek, nearly the whole of this portion of the face was destroyed. To conceal the deformity she has been in the habit of keeping her face tied up in a handkerchief; consequently, but little motion being allowed the lower jaw, this partial rest of the organ, persevered in for more than six years, has produced a permanent contraction of the masseter muscles on each side, so that scarcely any motion exists in the temporo-maxillary articulations, and it is impossible to introduce any substance more than the sixteenth of an inch in thickness between the upper and lower jaw. Her speech is of course very much impaired, and all her food is reduced to the smallest possible bulk, or taken in the shape of liquids: her general health is excellent.

"The first indication in such a case was obviously to obtain as much motion in the articulations of the lower jaw as possible; and this could only be accomplished by increasing the space between the maxillary bones. To accomplish this, it was deemed best to divide the masseter muscles (the entire muscle on the left, and what remained of it on the right side), and then separate the bones by a lever of some kind. Accordingly, on the first Wednesday in March, that being the regular clinical day at the College, she was brought before the class, and the operation performed with a common scalpel, the muscles being divided from *within,* and the knife carried obliquely downwards. The wounds were dressed with dry lint, and on the second day the lever of Heister was employed to separate the jaws. Each day the screw was turned a thread or two; and, after a lapse of two weeks, the patient was enabled to protrude her tongue without difficulty,—a thing utterly impossible when the treatment was commenced;—and the space between the teeth, when the lower jaw is depressed, is nearly an inch. She has, of course, free motion in the part, and chews her food without much difficulty.

"The most difficult part of the treatment still remained to be accomplished,

* This case is well illustrated by different sketches.

and on the 23d inst. she was again brought before the class, for the purpose of having this put into execution.

"After carefully considering the different operations usually performed in such cases, I adopted the following plan: Having first extracted the useless teeth of the upper jaw, which, from their irregularity, would have materially interfered with the proper adjustment of the flaps, and, besides, by their sharpness, possibly caused ulceration and sloughing of the tissues forced against them, I proceeded to detach the integuments by which the opening in the cheek was surrounded. The edge of the scalpel was directed towards the bone, and the incisions carried sufficiently far to allow the margins of the wound to be approximated to a considerable degree. This callous margin, formed of the 'inodular tissue,' was then pared off with a bistoury, in order to obtain, if possible, union by the 'first intention' between the flaps. An effort was then made to close the wound by sliding the detached integuments, from all sides, towards the centre, but they refused to yield, and it became necessary to make other incisions. [These are exhibited in a sketch, and consist of two crescentic cuts above and below the wound, which meet in the centre of either margin.] By these incisions *four flaps* were formed, and detaching them carefully from the subjacent parts, we found no difficulty in uniting them at a line which indicated the longest diameter of the opening. The twisted suture was employed, and the wound presented, after their introduction, the appearance exhibited in Fig. 3. [Here the crescentic incisions above mentioned are gaping, and the fresh-pared margins in accurate contact.] To support the whole, one or two straps were passed over the points upon which there was most strain, and over all a thin pledget of patent lint was laid, and the patient placed in bed. The hemorrhage was comparatively trifling, but few arteries requiring a ligature; and the operation, though painful and tedious, was borne out by the patient without a murmur." (Plastic Operations, p. 21 et seq.)

We do not think it necessary to follow the details of the subsequent treatment in this case. Of its entire success we are assured by the representation of the patient's face in the concluding sketch; and it certainly does great credit to Dr. Mütter's skill and ingenuity, whilst it speaks powerfully in favour of the 'opération par déplacement.'

IV. The lips. Of all the defects we have alluded to, no one is more unsightly to the beholders and distressing to the unhappy subject of it than loss of the lips. The importance of these organs in articulation, mastication, retention of the saliva, &c., renders the defect in question deserving of the best attention of those who profess and practise plastic surgery; and that this claim has been regarded, the numerous operations proposed, and the large share of letterpress devoted to cheiloplasty in the works before us, (especially M. Serre's,) sufficiently attest. Of the various causes which give rise to the loss or deficiency of the lips, we may enumerate the following as the most prominent: wounds, ulceration of a scrofulous, herpetic, syphilitic, mercurial or cancerous character; burns; and, though rarely, congenital absence or imperfection. On a previous occasion we noticed the antiquity of the cheiloplastic operation, which owed its origin to the mutilations practised in India on malefactors of various grades. Our German authors supply us with ample materials were we so disposed to dilate on this subject; but this our space will not admit of, and we regret that we cannot do justice, within reasonable compass to Von Ammon's modifications of the operations he first details. M. Serre has evidently paid unusual attention to this part of our subject, and we shall therefore draw more largely upon him, whilst we also notice what the talent of our transatlantic contemporary has contributed towards the perfecting of this branch of the art. In M. Serre's opinion, cheiloplasty is the most advanced of all the plastic operations on the face, and that its details may be subjected to more fixed and definite rules, to which the variations according to existing circumstances may be referred. Delpech appears to have been successful in the restoration of the lips, but laboured under the disadvantage of deeming it a 'sine quá non' to have mucous membrane in its proper place, to obtain which end he doubly-twisted his flap in some instances, and thus doubly endangered the vitality of the transplanted skin. "Give me mucous membrane" said he, "to cover the border of my new lip, and there is not a defect which I will not undertake to remedy." So near are we sometimes to the discovery of a simple truth, when we are misled by previously acquired prejudices. This desideratum, however, of Delpech, has been literally supplied by the ingenuity of M. Serre, the peculiarity and advantage of whose operation consists in the retention of the mucous surface, which is dissected up from the diseased structure, and reapplied on that which is brought from a distance to supply its place. He combats the natural objection to this mode of operating, that with the mucous membrane you probably also transplant the seeds of the disease you have been labouring to root out, by referring to facts, and the results of his experience; now as facts in surgery are quite as stubborn as in any other branch of learning, and as we are neither in a position nor willing to gainsay the results of M. Serre's experience, we cordially welcome this addition to our knowledge, and congratulate him upon his success. Of course the advocate of this plan admits that it is inapplicable where the mucous surface is positively involved in the disease; and we think it right to add that though M. Serre claims and deserves a considerable amount of credit for the more extended introduction of this method of coating a new lip, the principle has been recognized by Von Ammon and others, though not so fully carried out. The first case illustrative of his plan of treatment, was one of cancer which had almost entirely destroyed the lower lip, with the exception of the mucous membrane; the tumour was removed and the new lip obtained by raising a square flap of skin from the chin and neck into the gap, where it was fixed by sutures; and the ancient mucous membrane having been previously dissected up, accommodated itself to its new alliance.

In like manner case No. 2 succeeded, though the irregularity of the disease, (which involved the left commissure of the mouth and a part of the cheek,) rendered the operation more tedious and complex; but the third case exemplifies the dreaded risk of return of the disease (cancer;) one of the angles of the wound gave way, and before means could be taken for remedying the accident, malignant ulceration commenced, and the patient quitted the hospital uncured.* The narration of this unsuccessful case had restored us to good humour with our tetchy author, when our equanimity is again disturbed a few pages further on, by finding him involved in a warm dispute with one of his own countrymen, M. Sanson, who had stirred up the bile of the Montpellier surgeon by claiming priority for Dieffenbach, in the very operation we were in the act of discussing. As regards the *principle*, we again repeat that it has long

* The details of some of M. Serre's operations are difficult to render intelligible without the illustrative plates.

been recognized and acted upon by other surgeons, though we believe that M. Serre has a right to the merit of having extensively employed it in cheiloplasty; and, in proof of our assertion we cannot refrain from extracting a passage from our former article, though at the risk of being charged with conveying away the oyster for the benefit of a third party, whilst we leave the combatants a shell apiece; it is this: "We shall paraphrase our author's account of this operation, (for a new eyelid by Fricke;) premising that, when the condition of the conjunctiva is tolerably sound, it is dissected up from the remnants of the former lid, so as to be employed as a lining for the fresh one. Of course, the preexistence of any malignant disease, such as carcinoma, forbids this reservation, &c." This mode of operating was published by Fricke in 1829. But we must abstain from thus intermeddling with the disputes of others, and point our shafts of criticism for more useful occasions: before we leave the lower lip, we will fulfil our pledge of noticing a successful case of Dr. Mütter's, the plan of whose operation differs from that of most others that have been employed for similar deformities. The patient, a man 50 years of age, was the subject of a cancerous affection involving the entire lower lip, and the operation was performed in the following way:

"Having seated the patient in a favorable position, with his head supported against the chest of an assistant, I proceeded to the removal of the entire diseased mass, by a semi-elliptical incision, which started from the commissure of the mouth on one side, and terminated at a corresponding point on the other. From the centre of this line two slightly curved incisions [here there are references to a diagram,] were carried downwards and outwards, until they reached the base of the inferior maxillary bone. It is obvious that these incisions were separated from each (other) by a triangular piece of skin, the superior angle of which nearly reached the first incision. Then, from the terminal extremities of the (lower) incisions two others were carried upwards along the base of the lower jaw, until they reached a point opposite the initial and terminal points of the (first) incision. Two quadrangular flaps were thus marked out, and immediately detached from the subjacent bone. The hemorrhages having been arrested, and the patient allowed a few minutes of repose, the flaps were raised and placed in the position originally occupied by the lower lip, and there united to each other in the mesial line, and also by their lower thirds to the triangular piece of integument, by means of the twisted suture. By the elevation of these flaps a raw surface on each side was left to heal by the modelling process, or by granulation. The parts were dressed with the 'tepid water dressing,' the patient placed in bed, with his head elevated, and a rigid antiphlogistic system of treatment ordered. Nothing of interest in the subsequent management of the case presented itself; the parts healed kindly, and the patient recovered, without a trace of the disease remaining. More than two years have elapsed since the performance of the operation, and Mr. Lambert is perfectly well, and actively engaged in business." (pp. 31-2.)

This is a simple and nice operation, and preferable in our opinion, to the more serious incisions, &c., of Dieffenbach and Roux, (see our former article,) or the twisting of the pedicle of the flap, as practised by Liston. One remark naturally forces itself upon us here, which is that in this as in other cases there seems to be no inconvenience resulting from the absence of *real* mucous membrane, on the retention of which M. Serre lays so much stress: the simple explanation is that the surface which results from the healing process is so nearly allied to mucous membrane as to answer every purpose of that tissue; a fact which is but too familiar to surgeons in the analogous case of long-standing sinuses, the secretion of which so often becomes permanent, and of a mucoid character, requiring destruction of surface before a granulating process can be established.

In passing to the operation for renewal of the upper lip, M. Serre justly remarks that but little attention appears to have been given to it by authors who have treated of plastic surgery; in confirmation whereof we need merely to refer to the paucity of material which was afforded us in the compilation of the former paper devoted to this subject. We are therefore glad of the opportunity which our French author yields us of making good the deficiency in question, and shall accordingly avail ourselves of his cases to illustrate this division of our subject. The first case which is given in detail is one of considerable interest, and involving points of practice the importance of which are self-evident. The patient, a girl 17 years of age, was the subject of congenital double hare-lip, with division of the soft and hard palate, accompanied by great difficulty in deglutition and articulation. The incisive bone* and teeth, which were implanted in it, were very sensibly displaced in a direction forwards, as was also the fleshy median tubercle, which was elevated and directed forwards; the cleft of the palate was about three lines in diameter.

Before passing on to our author's operation in this case, we are tempted to notice a practical remark which he quotes from Dupuytren's 'Leçons Orales;' it has reference to the central appendage of skin which is remarked in these cases of double hare-lip. This excellent surgeon observes, "I have more than once had occasion to regret having left a deformity scarcely less than that for which I operated, by employing the central fleshy tubercle in the restoration of the lip.' This, it appears, necessarily results where the tubercle in question springs directly from the extremity of the nose; and the consequences attending these operations led M. Dupuytren to attempt the cure in the following way. Having separated the tubercle from its osseous attachment and dissected it up, it was raised horizontally and turned back so as to form a portion or the whole of the fleshy septum. The operation is then completed as in the case of a single hare-lip. It is only when the labial tubercle is inserted close to the osseous nasal spine that it should be retained as an integral part of the lip. But this by way of parenthesis.

M. Serre's operation may be epitomised thus: The two teeth which sprung from the "incisive bone" being removed, the greater part of the latter was likewise detached by two rectangular sections made with a pair of nippers. The next step was to appropriate the median lobule (which was attached in this instance to the extremity of the nose, and therefore useless for the lip,) to the formation of the septum; and this was effected by paring it to its proper size, and dissecting and turning it

* As this expression may not be familiar to all of our readers, we think it right to notice that it involves a point of questionable developmental anatomy. Béclard describes the superior maxillary bone as originally consisting of 1, a palatal; 2, an orbital and malar; 3, a nasal and facial; and 4, an incisor portion. The rapid ossification of this bone has prevented other anatomists from noticing this division; and Cruveilhier, amongst others, denies the separate existence of an incisive bone in man. It may be described as that portion of the palate which is immediately behind the incisor teeth, including the posterior margin of the alveolar ridge. We shall assume its existence.

back so as to be in readiness to be affixed to the surface of the section of the incisive bone. This being accomplished, the opposed edges of the lateral flaps were pared, and themselves separated, on a level with their junction to the gums, through their whole thickness, and to the extent of half an inch, with a view to facilitating their more ready approximation. The concluding step, before the application of the requisite ligatures, was a further separation of the natural adhesions between the retracted portions of the lips and the gums. In three weeks the patient left the hospital quite cured, "to the astonishment of all who had seen her before the operation, and unrecognized by the majority of her friends in her native place." M. Serre adds his regret that the palatal fissure was too wide to admit of reunion; "but the restoration of the lip had had the effect of materially improving the articulation." (pp. 152 et seq.)

The next case narrated occurred in the practice of M. Gensoul as early as 1830; and as we believe he was the first surgeon who carried into effect the modification by which it is characterized, we shall briefly refer to it. The patient was a young girl, 13 years of age, who had a congenital *double* hare-lip and cleft palate, as in the former instance; (we believe the latter is an almost invariable concomitant of the former;) there was further great prominence of the incisive portion of the superior maxillary bone, which projected beyond the lower jaw, and lateral portions of the upper to the extent of three quarters of an inch, so that the incisor teeth were directed horizontally forwards; lastly, the canine tooth and corresponding portion of jaw on the right side were likewise projecting forwards and outwards. The operation was as follows: The median flap was first dissected up, and held in this position by an assistant; the four incisor teeth were then withdrawn, and as the prominence of the bone was found so great as still to render the completion of the operation impracticable, M. Gensoul proposed, in the first instance, to excise the offending piece. But then, remarks our author, the patient would have been almost incapable of speaking without hissing, she would have been unable to spit out before her (!), and mastication would have been attended by inconveniences; under these circumstances the operator determined on the following plan: he seized the prominent bone with a powerful pair of forceps, and forcibly broke it and bent it back, the fracture extending to a level with the bicuspid teeth, and the fragment remaining suspended in its new relation by the palatal and pituitary membranes only. The right canine tooth was served in the same way without being removed: and the operation on the lip completed in the usual manner. The patient left the hospital in a fortnight, with the precautionary direction to confine herself to spoonmeat for a month; and the cure was ultimately complete. (Serre, pp. 161 et seq.)

In Dr. Mütter's pamphlet on 'Cleft Palate,' some observations occur, of which we may appropriately introduce a notice here. After classifying the congenital defects of the palate, according to their extent and the structures implicated, this surgeon remarks that where the whole palatine vault is divided, together with single or double hare-lip, he varies his management according to the age of the patient. "If called" he says, "a few days after birth, and the child is healthy, I operate for the hare-lip as soon as possible, believing as I do that the earlier the operation the better. Much needless dread of convulsions, sloughings, fevers, &c., exists in the minds of some, when they refer to operations of this kind upon very young children, but I have over and over again succeeded without the occurrence of an untoward symptom in infants of three, four, and five days old." Time, he thinks, is thus gained, the union taking place more rapidly, and the child prepared for its proper nourishment, no doubt a great desideratum; and (which is certainly a very interesting fact,) he has found "the influence exerted by the pressure of the cheeks and lips upon the maxillary bones, sometimes sufficient of itself to cause an entire closure of the fissure in the hard palate. The completion of the cure may then be left till the patient grows up. For projection of the maxillary bones he applies pressure with an instrument constructed on the principle of a truss, "the pad resting upon the bone, and the spring passing around the head, and so arranged as to retain its proper position without the risk of slipping:" a few days, or, at most, a few weeks will suffice to reduce the projection, and the ordinary operation may then be proceeded with. He has also found benefit from the employment (in very bad cases,) of a "small silver clamp, composed of two flat blades and a regulating screw;" for milder cases, the finger and thumb of the nurse may be introduced several times in the day, and the maxillary bones thus pressed together. The clamp has been found efficacious even at a few *years* old. The removal of the projecting maxillary bones by cutting forceps, Dr. Mütter deprecates, when it is practicable to accomplish the cure without it. Gensoul's operation he considers admissible and feasible in cases where the application of pressure would, from the age of the patient, be unvailable. In the comparatively rare cases of double palatine fissure he considers that very little can be accomplished by an operation, and that we must rely upon a plate of gold or platina for the closure of the openings in the palatine vault. (pp. 25 et seq.)

We regret we cannot find room for a detailed account of a satisfactory case under M. Serre's care in which a considerable cleft and deformity of the palate were remedied by continued pressure, exercised by means of "elastic levers," and constructed so as to press together the maxillary bones; but we must hasten to close this division of our subject by noticing the mode adopted by our author for restoring the upper lip after its removal for disease. One case was under treatment for fungus hæmatodes during three years, and after a series of operations was ultimately cured; but this is too long even to epitomise. In another case, a cancer occupying one half of the lip and extending to the nostril was removed, with the exception of the mucous lining and the free margin of the lip; a corresponding flap was then raised from the cheek on the same side, and after free separation from its surrounding connexions, was brought forward, (méthode par déplacement,) and fixed in its new position; after one or two little mishaps this case terminated satisfactorily. Another and still more extensive defect, resulting from the explosion of gunpowder, was remedied by a double flap which was united in the median line.

V. The eyelids. The restoration of the eyelids is to be distinguished from the simpler but very useful operations for ectropium and entropium, which were indeed introductory to the former. The extent of mischief of course varies considerably in these cases, from the partial loss of one lid to the total loss of both; and the causes of these defects may be classified under much the same heads as those which necessitate the reproduction of lips, viz., congenital deformity, disease, or injury. In our former article we devoted some space to this portion of our subject, and then had occasion to notice favorably the contributions of Von Ammon, who seems to have devoted much attention to the blepharoplastic operation, and has illustrated his treatment of the defects in question largely by reference to his own practice; but as there is little of novelty presented to our notice, we must pass the details of both our authors summarily by, merely pausing to notice that M. Serre seems to have applied his French method to the cure of these cases with equal success as to other departments of the plastic art: we must also do him the credit to say that, without the same reserve and jealousy we have had so often to rebuke in the foregoing analysis, he candidly yields the palm to German surgeons, in having given the first impulse to this division of plastic surgery. We are also forced, by the already extended character of our "supplementary" article, to pass by M. Serre's operation for "restoration of the lacrymal sac,"* and conclude by noticing (as we promised on a former occasion,) Dr. Mütter's successful operations for the removal of deformity consequent on burns, and for fissures of the palatine vault.

VI. Cicatrices from burns. The principle which guided Dr. Mütter in his operations on contracted cicatrices is not entirely new, though he appears to have been unusually successful in its application. The method is essentially plastic (we use the word in its restricted sense,) in its nature. The unvarying ill success which has attended the attempt to remedy the defect in question by simple section or excision of the offending texture, led Dr. Mütter to undertake the more complex operation of transplanting healthy skin to the position occupied by the cicatrix; and to our best belief he is the first author who has made public the result of his practice in these cases. Many of the deformities alluded to are, however, by no means of so simple a nature as to be remediable by operation on the skin alone; the neighbouring muscles, if not primarily involved, become so secondarily, by accommodating themselves to their altered relations; such, for instance, as the sterno-mastoid in contraction of the neck, the adductors of the humerus and flexors of the forearm in burns involving the upper extremity; and this is an especially formidable obstacle to restoration of the part when the injury has been received in childhood, and the applicant for relief has since grown to puberty. Nay more, we have witnessed, when all these difficulties have been surmounted, a further and most troublesome obstacle offer itself in the vessels and nerves of the limb. It therefore behoves the surgeon well to consider every possible difficulty he may have to encounter, before he undertakes an operation which may inflict much suffering, and entail permanent mischief on the patient, without in any degree remedying the defect for which the operation was undertaken.

We gave a short abstract of Dr. Mütter's first case in a recent Number of our Journal; we will now again notice it in connexion with his others. Miss A. T. had, it appears, been the subject of a severe burn on the

* In fact, it presents but little for notice or analysis; the mode of treatment recommended being the favorite one by displacement.

neck and throat when five years old, being upwards of twenty years prior to the date of the operation, 1841. Her life being despaired of at the time of the accident, she was permitted to lie in the position most agreeable to herself, and all medical interference was declined. As a necessary consequence the chin, cheeks, and face generally were drawn down and tied to the chest; the mouth was closed with great difficulty, and the right eyelid followed the skin of the face. But further there were other effects illustrating the power of a constantly operating cause in disturbing the normal growth of parts subjected to its influence: "the angles of the lower jaw were altered, and the incisor teeth nearly *horizontal*, by the pressure of the tongue, which organ, in consequence of the inability of the patient to close the mouth, was always visible, and indeed *protruded*, when she was silent." We have here copied the doctor's words, though we are disposed to question the justness of this explanation exclusively; the cause *we* alluded to was the traction of the skin attached to the jaw, which was, according to his showing, the reason why the mouth could not be closed; the pressure of the tongue probably aided secondarily to a trifling extent in producing the effect alluded to; though even this could not be the case when it was protruded, which was, it appears, its usual position. Still further, "the clavicle was so completely imbedded in the cicatrix, that it could scarcely be felt, and there was no external indication of its location. The chin, from the shortness of the bands, was drawn down to within *one inch and a half* of the top of the sternum, and the head consequently inclined very much. The space between the chin and sternum was also filled up by the cicatrix, so that no depression existed in front of her neck." Here indeed was a frightful condition, and one well calculated to test the ingenuity of the operator, and the value of the remedy. It would be difficult to do justice to the operation without transcribing the author's own account of it.

"The patient being placed in a strong light, and seated on a low chair, her head was thrown back as far as possible, and sustained in this position by an assistant. Seating myself in front, I began by making an incision, which commenced on the outside of the cicatrix in *sound skin*, and passed across the throat into *sound skin*, on the opposite side. This penetrated nearly through the integuments, and was made as near the centre of the cicatrix as possible. It was therefore about three-quarters of an inch above the top of the sternum, and of course in the most vital part of the neck. My object in making it so low down was to get at the attachments of the sterno-cleido-mastoid muscles, which, in consequence of the long flexion of the head, were not more than three inches in length, and required on one side *complete*, and on the other *partial* division, before the head could be raised. The integuments having been thus divided, I next carefully dissected through the cicatrix until I reached the fascia superficialis colli, which I could readily detect, and then going on still deeper, I exposed the sterno-cleido-mastoid muscles of the right side, and passing a director under it, as low down as possible, divided both its attachments. This enabled me to raise the head an inch or two, but finding that it was still kept down by the sterno-cleido-mastoid of the *left* side I divided the sternal attachment of this muscle, and was much gratified to find that the head could at once be placed in its proper position, the clavicular attachment of the muscle offering little or no resistance. A most shocking wound, *six inches in length*, by *five and a half in width*, was thus made, and yet there was scarcely any hemorrhage; three or four vessels only requiring the ligature. The next step in the operation consisted in the detachment of a flap of *sound skin*, with which this chasm could be filled; for I knew very well, that if permitted to heal by granulation only, the patient, so far from being benefited, would be made worse than before. To obtain this flap, I commenced at the terminal extremity of the first incision, and carrying the scalpel *downwards and outwards* over the deltoid muscle, dissected up an oval piece of integument *six inches and a half in length* by *six in width,* leaving it attached at the upper part of the neck. This dissection was painful, but not bloody, only one small vessel being opened. The flap thus detached was next brought round by making a half turn in its pedicle, placed in the gap it was destined to fill, and carefully attached by several twisted sutures, to the edges of the wound. Several straps were then applied to support the sutures and, with the exception of its upper third, was completely covered in. A pledget of lint, moistened with warm water, was laid upon this raw surface, a bandage applied, by which the head was carried backwards and maintained in this position, and the patient put to bed." (pp. 5 et seq.)

This bold operation was followed by the most satisfactory results, motion being restored to the head, the mouth closed, and the traction on the cheek and eye removed. A series of illustrative diagrams accompany the description, and the contrast between the first and last is indeed great. We may just add that nearly the entire margin of the flap adhered by the first intention in its new position.

The next case was in some respects even more unpropitious than that just related. The patient was a girl 12 years of age, and the accident likewise a burn. "For nearly eight years she had been unable to turn her head to the left side, the lower lip was everted, and the chin drawn down nearly in contact with the sternum, whilst the front of the throat presented a rough reddish cicatrix." The operation in this instance was nearly identical with the former, and the success at least equal, if we may judge by the accompanying sketches. In the third and last case the cicatrix occupied a more central position, causing the near approximation of the chin to the sternum, complete eversion of the lower lip, and obliquity in the direction of the lower incisor teeth; a similar mode of proceeding was followed by a similarly favorable result. One interesting feature in connexion with these operations is, that the constitutional disturbance was very trifling and transient, a fact one would scarcely have anticipated *a priori.* We congratulate Dr. Mütter on these highly creditable and acceptable contributions to practical surgery; and turn, in conclusion, to his pamphlet on fissured palate.

VII. FISSURE OF THE PALATE. There are few operations that have been long recognized and practised, in which surgeons have been oftener foiled than that for fissure of the palatine vault. The chief cause of this may perhaps be sought in the delicate texture of the membrane of the palate, and the necessary tension which accompanies the approximation of the edges of the cleft. Dr. Mütter's success, however, appears considerably to exceed that which has fallen to the lot of others; for we find him stating at the commencement of his paper, that "out of twenty-nine operations on the soft and hard palate which he has performed, he has failed to relieve the patient but in two cases; and that he has met with no dangerous symptoms in any case." This is, we think, unprecedented success; and we shall be doing our surgical readers good service by detailing the steps of our author's course of proceeding,—not, however, exactly holding his opinion that "reports of rare and successful cases are more for the benefit and instruction of the very young, than for the information of the older surgeons." He divides his operation into the usual stages of "denudation of the edges of the fissure, introduction of the ligatures, and approximation of the edges, and tying the ligatures;" and deprecates the practice recommended by some, of introducing the ligatures before the incisions are made,—the hemorrhage is easily arrested, he adds, by causing the patient to gargle with cold water. In freshening the edges, it is recommended to commence at the most dependent point; for, by making the section from below upwards, the blood will not obscure the parts. Under the several heads of "age, health, season, preparation of the patient, &c.," the following remarks occur. It is hardly safe to undertake the operation before the sixteenth or eighteenth year. The general health of the patient should be favorable, and he should be free from any disorder of the assimilative functions, and from any specific disease. The most favorable season is that which is least liable to atmospheric vicissitudes; the occurrence of cold or cough being disastrous to the operation. As a preparative step, Dr. Mütter has found much benefit result from "frequent introduction into the fauces and between the edges of the cleft, of the instrument to be used, or the finger of the surgeon or of the patient himself, by which the parts become, as it were, *familiarized* to the presence of foreign bodies." As a favorable effect of this precaution, in neither of the cases narrated in the pamphlet was it found necessary to introduce any foreign body to prevent the patients' mouth from closing, or their interfering by motion of the tongue. The needles should be well tempered to prevent their breaking, which has occurred, and the fragments have fallen into the œsophagus. The great after-difficulties are those which attend the desire for food, and especially the thirst; our author does his best to keep his patients without food for three, or two days at least, and then allows thin calf's-foot jelly or "what is known as cold custard or slip;" of this last article we do not know the British synonym. To allay the thirst the patient may be allowed the luxury of a wet sponge brought in contact with the roof of the mouth, or, at most, a teaspoonful of water "may be allowed occasionally to trickle down the throat;" this indeed requires no small share of philosophy. Again, any disposition to cough or sneeze must be scrupulously avoided, as tending, from the accompanying spasm, to interfere with the new relation of the parts; and the patient must be guarded against the desire to "clear the throat," which naturally springs from the irritation of the fauces. But now for the case.

The patient was a gentleman 25 years of age, in excellent health and very anxious for relief. The fissure, which was congenital, extended through the centre of the soft palate, and involved the posterior third of the palate bones. After the "preparation" noticed above, the patient was placed in a chair, and his head firmly supported against the chest of an assistant. The first step in the operation consisted in an assistant "inserting a sharp hook into the most depending angle of the left margin of the cleft," by which means the parts were made tense.

"I then inserted," proceeds Dr. Mütter, "the point of a thin *double-edged knife*, (the blade of which was one inch, and the handle six inches in length,) in the most dependent part of the margin, about a line from its free edge, and cut rapidly from *below, upwards*, inclining the knife so as to reach the apex of the cleft. When the apex was reached, the knife was changed from the right hand to the left, and Dr. Randolph (the assistant) passing the hand which held the hook, across and a little above the face of the patient, made pretty firm traction upon the slip of mucous membrane previously separated by the first cut, and which still remained transfixed by the hook; by this means the right margin was made tense. I then completed the denudation by cutting rapidly from above, downwards. The denudation of the margins occupied about a minute, and the patient was then allowed to rest. The hemorrhage was slight and easily controlled by gargling with cold water, and after the lapse of a few minutes the second step of the operation was commenced . . . The head being placed and firmly supported as before, and the mouth held open by the volition only of the patient, I passed a small curved needle, armed with a well-waxed double silk ligature, and firmly held in the grasp of Physick's forceps,* through the most dependent part of the left margin of the cleft, carrying the needle from before backwards, and inclining my hand to the left of the mouth, so as to throw the point of the needle, after it had transfixed the tissues, into the middle of the cleft. As soon as it was visible, Dr. Randolph seized it with a pair of long forceps, and the clamp of the porte being at the same time relaxed, by which the grasp upon the needle was kept up, the latter was loosened and at once withdrawn from the mouth. The same needle was immediately replaced in the porte, and the latter being held in the right hand, instead of the left, I introduced the needle on the right margin of the fissure, at a point as nearly opposite as possible the little wound in the left, passing it from behind forward. As soon as its point was visible, it was seized and drawn through, and thus the first ligature was passed. The patient was then allowed to rest for a few minutes, and then the second ligature was passed in the same manner; a third and a fourth were also required, and between the introduction of each there was a respite allowed, during which the patient gargled with cold water, took a little wine and water, and had the blood mopped away. The whole were passed in about fifteen minutes, and as the needles were detached from each, their extremities were carried out at each corner of the mouth, and held separate by assistants. The needles were all introduced from two to three lines from the margins. The third and last step was then undertaken, and we commenced it by tying the ligature first introduced, or that nearest the uvula. The first knot was easily made by crossing the ends of the ligature, wrapping them round the ends of the fore-fingers of each hand, and then passing the fingers as far back as possible. The edges came together beautifully, and with but little strain by slowly carrying the fingers outward, and then the second knot was made. While the ends were crossing for this knot, Dr. Randolph grasped the first with a pair of forceps, and held it until the second was completed. Lastly, the ends of the ligatures were cut off close, and the patient allowed to rest for a few minutes. The others were knotted in the same manner, and in thirty-five minutes the patient was in bed." (p. 3, et seq.)

We should have found it difficult to curtail the above case (albeit it possesses nothing strikingly novel or peculiar in its details,) without depriving it of the interest and value which it derives from the mode in which the different steps were conducted,—an interest which is so much enhanced by the fact already alluded to, of its being one of the nineteen successful cases in twenty-one. The only instruments, apparatus, &c., required consisted of " a knife, a hook, a pair of long forceps, a simple porte and needles, with waxed ligatures, scissors, sponges on handles, wine and water, cold water, towels, and two or three assistants." The only drawbacks in the subsequent course of the case were the giving way of one ligature and a tendency to slough at the spot: this was met by caustic solutions. The other ligatures were taken away on the fifth, sixth, and seventh days, and the cure (with the exception of a

* A long, slightly curved, pair of forceps, which close with a spring.

pin-hole opening,) was complete at the end of three weeks. The usual long duration of these operations (as compared with the above) Dr. Mütter attributes to the complexity of the instruments employed. One or two remarks on the after treatment we may append with advantage. Our author has often observed severe griping distress his patients; this he attributes to the quantity of blood swallowed during the operation, and has found the best remedy to be an enema repeated every hour till the blood is brought away, or, if requisite, an anodyne injection. If inflammation of the fauces run high, the most active antiphlogistic treatment is to be adopted, lest it should terminate in sloughing, or an extension of the inflammation to the lungs. Fatal cases have resulted from these causes, though they are rare. When the pared edges cannot be readily approximated Dr. Mütter approves of lateral incisions, as proposed by Dieffenbach to relieve the tension. An ingenious mode of treating small apertures in the palate is mentioned, and illustrated by two cases; the plan consisting in a combination of sliding the flap and of granulation; but for the particulars we must refer our readers to the original pamphlet, or the paper in the American Journal of Medical Sciences.*

* As the valuable and very interesting paper on cleft palate, read by Mr. Fergusson, at the close of last year, before the Medico-Chirurgical Society, has not yet appeared in an authenticated form in print, it would be clearly beyond our province, as reviewers, to take any lengthened notice of it. It would, however, be injustice alike to the author of the paper and to our subject, if we did not furnish our readers with such a brief view of Mr. Fergusson's principles and practice in this case, as we can command; more especially as we regard the mode of operating proposed, though on a small scale, as one of the most ingenious and happiest applications of anatomical and physiological science to the art of surgery, that we have met with of late years. We take the following report of the contents of Mr. Fergusson's paper from the weekly journals of the day:

"The author commences his paper by making some general remarks on the operations for cleft palate performed in this country and abroad. He then proceeds to give a detailed account of a dissection which he had the opportunity of making, of the muscles which operate upon the soft palate, in an individual who had both the velum and a portion of the hard palate cleft. This description is followed by an examination of the opinions of different eminent physiologists, concerning the motions of the velum palati and its arches during the acts of deglutition, and by the author stating his own views as to the actions of the various muscles when the palate is cleft. This part of the subject he further illustrates, by describing four different states in which the flaps on each side may be seen, upon looking into the mouth of a person who has a cleft palate, and irritating them in different ways. By pursuing this course of anatomical and physiological enquiry, he arrives at the following conclusions: 1, that the flaps are slightly drawn upwards and to the sides, when the levator palati contracts; 2 that when the levator palati and palato-pharyngeus act strongly and together, the flaps are so forcibly drawn from the mesial gap, that they can scarcely be distinguished from the sides of the pharynx; 3, that the flaps are forced together, and the edges come into contact, when the superior constrictor muscle contracts during the act of deglutition; 4, that the circumflexus palati possesses but a feeble power over the flaps; lastly, the fibres of the palato-glossus were very imperfectly developed in the specimen in his possession. The chief object of his paper is to communicate a novel plan of operating in staphyloraphy, founded on the above investigations, and which he has put in practice with most satisfactory results in two cases during the last twelve months. The principle of his new proposal is to divide those muscles of the palate which have the effect of drawing the flaps from each other, and widening the gap between them when they contract, so that the stretched velum may be in a state of repose, and the joined edges may not be pulled asunder by any convulsive action of the parts during the process of union. In other words, he advises, as an accessory to the operation of staphyloraphy, the division of the levator palati and palato-pharyngeus muscles; and, if requisite, the palato-glossus. In bringing forward this plan, he reviews the different modes of operating which have been pursued by numerous distinguished surgeons who have written on the subject; and he concludes by entering into several

We have but little to add as a parting word to our different authors. Von Ammon's and Dr. Baumgarten's joint work possesses the characteristics common to most specimens of German medical literature, that of presenting a well arranged and sufficiently copious view of the branch of practice to which it refers. In spite of the morbid susceptibility and reclamatory style of M. Serre, which have so frequently called for our friendly criticism, there is much of simplicity and even candour which has been an agreeable relief to us in perusing his volume: of his merits as a surgeon we have a high opinion, and only wish we could persuade him, "et id genus *irritabile* omne," that it is by no means the surest mode of securing the public verdict in their favour, to be over-solicitous about their own fame, which is very apt to make their critics ill-natured, and their readers suspicious; but we are aware that we must set down much to the spirit of "la gloire de la grande nation," which burns in every Frenchman's bosom, and is so ready to burst forth into a consuming flame when stirred by national slights. Dr. Mütter's pamphlets are simple and, we may add, modest records of a success which we cannot quarrel with him for being justly proud of.

---

### ART. VII.

minute details regarding the steps into his own operation, and by describing the particular forms of instruments which he has found best adapted for his proceedings.

The preparation of cleft palate, a dissection of the parts in the usual condition of the throat, a variety of diagrams, instruments, &c. were on the table, to illustrate the views of the author." (Medical Times, Dec. 21, 1844.)

